# Combined therapy with ceftriaxone and doxycycline does not improve the outcome of meningococcal meningitis in mice compared to ceftriaxone monotherapy

**DOI:** 10.1186/s12879-020-05226-w

**Published:** 2020-07-13

**Authors:** Susanna Ricci, Denis Grandgirard, Ilias Masouris, Tiziana Braccini, Gianni Pozzi, Marco R. Oggioni, Uwe Koedel, Stephen L. Leib

**Affiliations:** 1 Department of Medical Biotechnologies, Laboratory of Molecular Microbiology and Biotechnology (LA.M.M.B.), Ospedale Santa Maria alle Scotte, University of Siena, Siena, Italy; 2 ESCMID Study Group for Infectious Diseases of the Brain (ESGIB), Basel, Switzerland; 3 Institute for Infectious Diseases, University of Bern, Bern, Switzerland; 4 Department of Neurology, Ludwig-Maximilians University, Munich, Germany; 5 Department of Genetics and Genome Biology, University of Leicester, Leicester, UK

**Keywords:** *Neisseria meningitidis*, Meningococcal meningitis, Mouse model, Doxycycline, Adjunctive therapy, Brain damage, Matrix metalloproteinases

## Abstract

**Background:**

Meningococcal meningitis (MM) is a life-threatening disease associated with approximately 10% case fatality rates and neurological sequelae in 10–20% of the cases. Recently, we have shown that the matrix metalloproteinase (MMP) inhibitor BB-94 reduced brain injury in a mouse model of MM. The present study aimed to assess whether doxycycline (DOX), a tetracycline that showed a neuroprotective effect as adjuvant therapy in experimental pneumococcal meningitis (PM), would also exert a beneficial effect when given as adjunctive therapy to ceftriaxone (CRO) in experimental MM.

**Methods:**

BALB/c mice were infected by the intracisternal route with a group C *Neisseria meningitidis* strain. Eighteen h post infection (hpi), animals were randomised for treatment with CRO [100 mg/kg subcutaneously (s.c.)], CRO plus DOX (30 mg/kg s.c.) or saline (control s.c.). Antibiotic treatment was repeated 24 and 40 hpi. Mouse survival and clinical signs, bacterial counts in cerebella, brain damage, MMP-9 and cyto/chemokine levels were assessed 48 hpi.

**Results:**

Analysis of bacterial load in cerebella indicated that CRO and CRO + DOX were equally effective at controlling meningococcal replication. No differences in survival were observed between mice treated with CRO (94.4%) or CRO + DOX (95.5%), (*p* > 0.05). Treatment with CRO + DOX significantly diminished both the number of cerebral hemorrhages (*p* = 0.029) and the amount of MMP-9 in the brain (*p* = 0.046) compared to untreated controls, but not to CRO-treated animals (*p* > 0.05). Levels of inflammatory markers in the brain of mice that received CRO or CRO + DOX were not significantly different (*p* > 0.05). Overall, there were no significant differences in the parameters assessed between the groups treated with CRO alone or CRO + DOX.

**Conclusions:**

Treatment with CRO + DOX showed similar bactericidal activity to CRO in vivo, suggesting no antagonist effect of DOX on CRO. Combined therapy significantly improved mouse survival and disease severity compared to untreated animals, but addition of DOX to CRO did not offer significant benefits over CRO monotherapy. In contrast to experimental PM, DOX has no adjunctive activity in experimental MM.

## Background

Meningitis is an inflammation of the meninges and subarachnoid space that may also involve the cerebral cortex and parenchyma. *Neisseria meningitidis* is the second most common cause of bacterial meningitis (BM) after *Streptococcus pneumoniae* worldwide [1]. About 10–30% of the human population asymptomatically carries the meningococcus in the nasopharynx, which also represents the first step for transmission and onset of invasive meningococcal disease with infants, children and adolescents being the groups at higher risk [2]. The most frequent clinical presentation of invasive disease is meningococcal meningitis (MM) [2], which can affect 30–50% of patients [3, 4]. MM is characterised by 7–18% case fatality rates depending on meningococcal serogroup and patient age [3, 4]. Long-term physical, neurological and psychological sequelae may occur in up to 20% of survivors [5], of which about 7% suffer from neurological sequelae (i.e., hearing loss, cognitive impairment, motor deficits, seizures) [6].

Both the host inflammatory and immune response to infection and the direct cytotoxicity of bacterial factors contribute to brain damage [7–9]. Histopathological studies in humans report different forms of brain injury associated with BM, including brain edema, hydrocephalus, cortical necrosis, petechial hemorrhages, loss of myelinated fibers in the white matter, hippocampal apoptosis, and injury to the inner ear [1, 7–9]. There are numerous mediators of brain damage, including matrix metalloproteinases (MMPs). MMPs are proteolytic enzymes which can act as both proteinases by degrading extracellular matrix components and convertases by activating cytokines and their receptors [10]. Therefore, MMPs play a pivotal role in the pathogenesis of BM and brain injury by promoting inflammation, disruption of the blood-brain barrier (BBB), polymorphonuclear (PMN) cell extravasation and tissue destruction [11, 12]. MMPs are elevated in the cerebrospinal fluid (CSF) of patients with BM [13–16], and high levels of MMP-9 are significantly associated with increased risk of neurological sequelae [15] and death [16]. In experimental models of BM, MMPs have been shown to contribute to brain damage [17–19], and pharmacological inhibition of MMPs was effective at increasing animal survival and reducing both cortical injury and hippocampal apoptosis (reviewed in [12]). Amongst the MMP inhibitors tested, doxycycline (DOX) is a second generation tetracycline able to penetrate into the CSF and equipped with both antimicrobial activity and broad anti-inflammatory properties [20], including inhibition of MMPs and tumor necrosis alpha converting enzyme (TACE) [21]. Adjunctive therapy with DOX in combination with ceftriaxone (CRO) reduced mortality and injury to the brain and cochlea in infant rats infected with *S. pneumoniae* [22].

Over the past 20 years, preclinical studies on adjunctive therapy in BM have mainly focused on experimental models of pneumococcal meningitis (PM) [23]. In contrast, as humans are the only natural hosts for *N. meningitidis*, hardly any work has been carried out in animal models of MM. Nevertheless, a MM experimental model based on intracisternal (i.c.) infection has been developed in adult mice [24]. Evaluation of brain damage by histology and bacterial immunostaining showed the typical traits of BM, including meningeal and ventricular inflammation, vasculitis, bleeding and hippocampal apoptosis together with meningococcal localization on the meninges, in the ventricles, in the choroid plexus and also in the *corpus callosum* of infected mice [24, 25]. The above findings resemble the features of meningococcal meningoencephalitis in humans, as described in clinical and autopsy studies of patients with MM [26–31]. The mouse model was recently used to assess the impact of batimastat (BB-94), a broad MMP inhibitor, on MM-associated cerebral injury [32]. Results showed that BB-94 significantly reduced cerebral bleeding and BBB breakdown, suggesting that MMPs contribute to the pathophysiology of MM and brain damage [32].

In the present study, we have assessed the efficacy of DOX given as adjunctive therapy together with CRO in experimental MM. Results showed that, although mice treated with CRO + DOX had increased survival and reduced brain injury compared to untreated controls, combined therapy offered no significant advantages compared to CRO monotherapy.

## Methods

### Meningococci and growth conditions

The serogroup C 93/4286 isolate of *N. meningitidis*, belonging to the ET-37 hypervirulent lineage, was kindly provided by Paola Salvatore, Naples, Italy. Bacteria were cultured at 37 °C in 5% CO_2_ on chocolate GC agar (Oxoid, Milano, Italy) or CG broth (Oxoid) added with 1% (v/v) Vitox, a culture medium supplement of essential growth factors (Oxoid). Inocula for mouse challenge were prepared by cultivating bacteria in GC broth until they reached an optical density at 600 nm (OD_600_) of 0.8–1.0 corresponding to the early/mid-logarithmic phase of growth. To determine CFU counts, an aliquot of bacterial cultures was serially diluted and plated (approximately 10^9^ CFU/ml). Meningococci were finally frozen at − 80 °C with 10% glycerol until use.

### Mice

Six-weeks-old female BALB/c mice weighing 18–20 g were purchased from Charles River (Calco, Italy). No written permissions were required. Animals (5–8 mice/cage) were housed in filter top cages (Tecniplast S.p.a, Varese, Italy) in a ventilated cabinet with a controlled temperature of 20–24 °C and 12 h light/dark cycles. Mice were given food and water ad libitum and allowed to settle in the new environment for 1 week before performing the experiments. Animal experiments were authorised by the local ethics committee ‘Organismo Preposto al Benessere Animale’ (document no. 26094-X/10) and the Italian Ministry of Health (document no. 815/2015-PR and following amendments), and were carried out according to institutional guidelines. The study was conducted in accordance with the ARRIVE guidelines for reporting animal experiments.

### Model of MM-induced brain damage

The model was developed based on a previously described MM mouse model [24]. Briefly, 2 h before meningococcal challenge, animals were treated with an intraperitoneal (i.p.) injection of iron dextran (250 mg/kg; Sigma-Aldrich, Milano, Italy). Bacteria were thawed, washed, and suspended in GC broth with iron dextran (5 mg/kg) at a final concentration of 10^8^ CFU/ml. Mice were lightly anaesthetised by i.p. injection with Zoletil [(tiletamine and zolazepam hydrochloride), 15 mg/kg; Virbac Srl, Milano, Italy] and Xilor [(xylazine 2%), 4 mg/kg; Bio 98 Srl, Milano, Italy] and infected intracisternally (i.c.) with 10 μl of the bacterial inoculum (10^6^ CFU/mouse corresponding to a lethal dose killing 20% of animals, LD_20_). Mice were closely monitored, and clinical signs were recorded according to a coma scale described for rodents [33]. Animals were humanely sacrificed if/when they reached a score of 2.

### Experimental design

Based on a previously published study of ours [32], 3 independent experiments (18–19 mice/experiment) were performed with a total of 55 mice (control, *n* = 15; CRO, *n* = 18; CRO + DOX, *n* = 22). Animals were infected by the i.c. route as described above and randomised for antibiotic treatment 18, 24 and 40 h post infection (hpi). Mice were injected subcutaneously (s.c.) with CRO (Fidia Farmaceutici S.p.A, Abano Terme, Padova, Italy; 100 mg/kg) or CRO plus DOX (Calbiochem, Merck Millipore, Merck KGaA, Darmstadt, Germany; 30 mg/kg). Antibiotic treatment was according to Meli et al. [22]. Animals treated with both drugs received DOX first and CRO after 15 min. Control mice were injected with phosphate buffered saline (PBS). Forty eight hpi, animals were lightly anaesthetised as described above and then sacrificed by i.p. injection with an overdose of Zoletil (30 mg/kg; Virbac Srl) and Xilor (8 mg/kg; Bio 98 Srl). Cervical dislocation was employed on all euthanised mice prior to sample collection.

### Sample collection

Brains and cerebella were collected. Brain samples were immediately frozen in dry ice for assessment of cerebral bleeding. Cerebella were split into halves, of which one was used for CFU counts and the other was frozen in dry ice for MMP, and cyto- and chemokine analysis. Samples were not collected from animals found dead or sacrificed before 48 hpi.

### Bacterial counts in cerebella

Half cerebellum from each mouse was homogenised in 1 ml of Brain Heart Infusion (BHI; Oxoid) with 10% glycerol by using a Falcon® 70 μm cell strainer (BD Biosciences, Milano, Italy). Samples were serially diluted in BHI and plated onto chocolate CG agar plates to determine CFU counts/cerebellum. Assay detection limit was 20 CFU/cerebellum.

### Quantification of cyto- and chemokines

A panel of cyto- and chemokines (TNF-α, IL-1β, IL-6, IL-10, MIP-1β, MCP-1, IP-10, KC, TIMP-1) was quantified in cerebellum homogenates by using microsphere-based multiplex assays (Milliplex MAP mouse cytokine/chemokine magnetic bead panel, Merck Millipore, Billerica, MA, USA). Cerebella were homogenised and treated as described before [32]. The total protein content of samples was determined by the BCA protein assay (Pierce, Thermo Fischer Scientific, Reinach, Switzerland). One hundred μg of homogenate were tested in duplicate, and at least 50 beads/analyte were measured using a Bio-Plex 200 station (Bio-Rad Laboratories, Hercules, CA, USA). Calibration curves from recombinant standards were calculated with the Bio-Plex Manager software (version 4.1.1) using a five-parameter logistic curve fitting.

### Gelatin-sepharose affinity binding and gelatin zymography

Gelatin affinity binding and zymography were performed as previously reported [32]. Briefly, gelatinases were enriched by incubating 100 μg of brain homogenate (see previous section) with 20 μl of Gelatin Sepharose 4B (GE Healthcare GmbH, Glattbrugg, Switzerland). Sepharose beads were washed, incubated with 2X zymography sample buffer, and centrifuged to elute bound proteins. Eluted proteins were subjected to electrophoresis under non-reducing conditions in polyacrylamide gels containing 1% (v/v) type A gelatin from porcine skin (Sigma-Aldrich). After electrophoresis, the MMP catalytic sites were activated in zymography buffer, and gels were finally stained as described [32]. Gelatinolytic activity was assessed by densitometric quantification of the substrate lysis zones around 92 kDa (pro-MMP-9) using the ImageJ analysis software [34]. Purified human neutrophil MMP-9 (Calbiochem) was used as a standard for normalization and quantification of MMP-9 as a function of the lysis zone.

### Analysis of cerebral bleeding

Brain hemorrhages were analysed as previously described [35]. Briefly, brains were cut in a frontal plane into 30 μm-thick sections, and serial sections were photographed with a digital camera at 0.3 mm-intervals. For each animal, the number of bleeding spots was counted on 5 comparable brain sections.

### Statistical analyses

Statistical analyses were performed using GraphPad Prism (Prism 7, GraphPad Software Inc., San Diego, USA). Results are presented as mean ± standard deviation (SD). Differences in clinical signs, cerebral bleeding, MMP-9 gelatinolytic activity and inflammatory mediators amongst the 3 mouse groups (CRO, CRO + DOX, control) were analysed by using the non-parametric Dunn’s multiple comparison test (*p* < 0.05). Mouse survival was evaluated by the Kaplan-Meier survival analysis, and differences were compared using the log-rank test (*p* < 0.05).

## Results

The effect of treatment with adjunctive DOX on MM outcome was investigated 48 hpi by assessing bacterial viable counts in cerebella, mouse survival and clinical signs, brain damage, and levels of MMPs and cyto- and chemokines.

### Impact of treatment with adjunctive DOX on animal survival and clinical signs

**Fig. 1:**
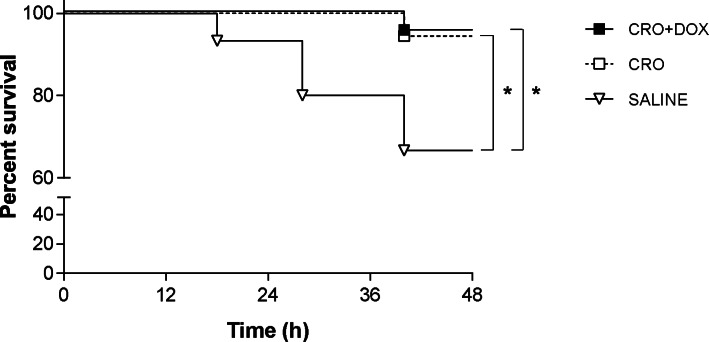
Survival of mice infected with meningococci and treated with CRO + DOX. Animals were infected i.c. with *N. meningitidis* and randomised for treatment with CRO (*n* = 18), CRO + DOX (*n* = 22) or saline (controls, *n* = 15) 18 and 40 hpi. Mice were sacrificed 48 hpi. Survival curves were compared by the log-rank test. Asterisks indicate statistical significance (*, *p* < 0.05)

Upon sacrifice 48 hpi, the clinical score of untreated, surviving mice was 2.7 ± 0.4 (*n* = 10). Antibiotic therapy significantly improved the clinical condition of animals that had received CRO (3.5 ± 0.5, *p* = 0.0018; *n* = 17) or CRO + DOX (3.4 ± 0.6, *p* = 0.0062; *n* = 21) compared to untreated controls, but no differences were found between the groups treated with CRO or CRO + DOX (*p* > 0.05). Kaplan-Meier analysis showed that the survival percentages of animals treated with CRO or CRO + DOX were 94.4 and 95.5, respectively, while survival of control mice was 66.7% (Fig. 1). As observed above with the clinical scores, differences in survival between animals treated with CRO (*p* = 0.036) or CRO + DOX (*p* = 0.017) and untreated controls were significant, but again no differences were calculated between mice treated with CRO or CRO + DOX (*p* > 0.05) (Fig. 1).

### CRO and CRO + DOX are equally effective at killing meningococci in the brain

CFU counts in cerebella of untreated mice (*n* = 10) 48 hpi were 2.5 × 10^6^ ± 7.6 × 10^6^ (mean ± SD). Bacterial titers in animals treated with CRO were 3.3 × 10^2^ ± 7.8 × 10^2^ (*n* = 17), and CFU counts were below detectable levels (20 CFU/cerebellum) in 12 out of 17 mice (data not shown). Combined therapy with CRO and DOX (1.6 × 10^2^ ± 5.5 × 10^2^; *n* = 21) was as effective as CRO at meningococcal killing, and 15/21 cerebella were found cleared from infection (data not shown). The result suggests no antagonistic effect of DOX on CRO.

### CRO + DOX combined therapy is not significantly more effective than CRO monotherapy at reducing cerebral bleeding in mice with MM

**Fig. 2:**
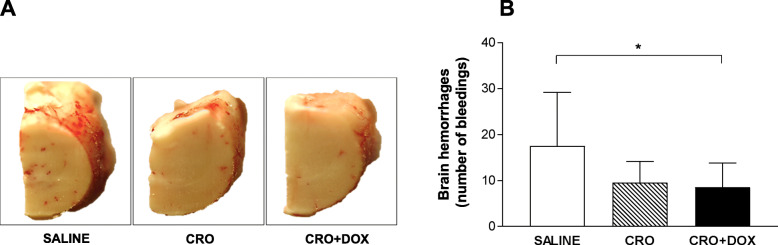
Effect of treatment with CRO + DOX on brain bleeding. Experimental design is as reported in Fig. 1. Brain hemispheres were collected 48 hpi, cut in 30 μm cryosections, and photographed with a digital camera to determine the number of bleedings. At least 5 slices/mouse were examined. **a** Macroscopical assessment of brain hemorrhages. Photos from a representative experiment out of the 3 performed is shown. **b** Enumeration of cerebral hemorrhages in animals treated with CRO (*n* = 17), CRO + DOX (*n* = 21), or saline (*n* = 10). Differences were analysed by the Dunn‘s multiple comparison test (*, *p* < 0.05)

Previously published results proved cerebral bleeding as a marker of MM-associated brain injury in our mouse model [32]. Therefore, the number of intracerebral hemorrhages was counted in the three animal groups. Results evidenced a pronounced reduction in the number of bleedings in mice treated with CRO (9.6 ± 4.6; *n* = 17) or CRO + DOX (8.6 ± 5.2; *n* = 21) compared to untreated controls (17.5 ± 11.7; *n* = 10), (Fig. 2). Although this reduction reached statistical significance only for animals administered with CRO + DOX compared to controls (*p* = 0.029), no significant differences were observed between animals treated with CRO and CRO + DOX (*p* > 0.05) (Fig. 2).

### Adjunctive DOX has no anti-inflammatory activity in the MM model

**Fig. 3:**
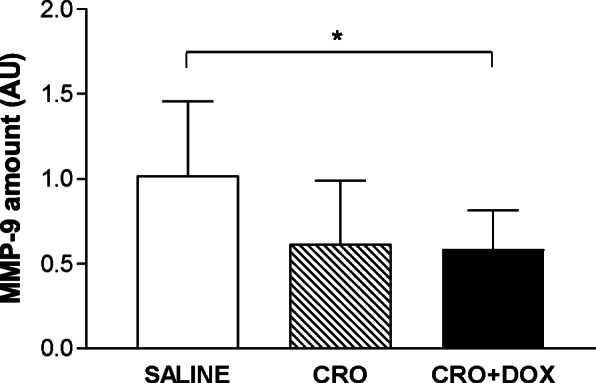
Gelatin zymography on cerebella of infected mice treated with CRO + DOX. Animals were infected with meningococci and treated as described in Fig. 1. Cerebellum halves were homogenised, incubated with gelatin sepharose and subjected to gelatin SDS-PAGE zymography to determine the amount of MMP-9. Human MMP-9 was used as a standard. Results from 3 independent experiments are shown. Data were analysed by the Dunn’s test (*, *p* < 0.05). AU, arbitrary units

DOX has been shown to have broad anti-inflammatory properties, including inhibition of MMPs and reduction of cytokine release [21]. To evaluate whether DOX inhibited MMP-9, gelatin zymography was performed on protein extracts from the cerebella of CRO, CRO + DOX treated and untreated animals 48 hpi. Densitometric analysis of MMP-9 substrate lysis zones showed a reduction of MMP-9 amount in rodents treated with CRO (0.61 ± 0.4; *p* = 0.06; *n* = 17) and CRO + DOX (0.58 ± 0.2; *p* = 0.046; *n* = 21) in comparison with controls (1 ± 0.4; *n* = 10), (Fig. 3). Statistical significance was observed only for the group treated with CRO + DOX. However, likewise the results on brain bleeding, no significant differences in MMP-9 levels were found between animals subjected to monotherapy and those that received the combined therapy (*p* > 0.05), indicating no adjunctive activity of DOX on CRO.

The impact of DOX on the inflammatory response was assessed by Luminex quantification of a set of inflammatory mediators in mouse cerebella. Treatment with CRO diminished inflammation, and differences with untreated mice were significant for TNF-α (*p* = 0.024) and IL-10 (*p* = 0.0026), (Table 1). No significant differences were observed between animals that received CRO alone and those subjected to CRO + DOX therapy.

**Table 1:** Quantification of inflammatory mediators in cerebella of infected mice

Group ^a^	Analyte (pg/ml) ^b^								
	TNF-α	IL-1β	IL-6	IL-10	MIP-1β	MCP-1	IP-10	KC	TIMP-1
**Saline**	4.2 ± 2.5	287 ± 48	1283 ± 1261	10.5 ± 7.1	478 ± 260	4317 ± 4082	357 ± 208	1991 ± 1312	39,689 ± 22,705
**CRO**	1.5 * ± 0.8	246 ± 66	100 ± 107	2.5 ** ± 2	132 ± 108	777 ± 1130	227 ± 144	478 ± 196	21,273 ± 12,715
**CRO + DOX**	2.7 ± 1.5	249 ± 70	1182 ± 1273	3.9 ± 1.6	510 ± 370	4322 ± 4373	687 ± 572	2218 ± 2426	25,617 ± 10,617

^a^Mice were divided in 3 groups based on treatment: saline (control), CRO or CRO + DOX

^b^refer to the results of statistical analysis

*/**, statistically significant differences compared to control mice (saline); *, *p* < 0.05; **, *p* < 0.01

## Discussion

Mortality and neurological sequelae due to BM occur as a consequence of systemic and intracranial complications. In the last two decades, it became clear that a combination of antimicrobial and adjunctive therapy would represent the most desirable approach to fight the pathogen as well as control the overwhelming host response to infection [1, 8, 36]. Preclinical studies in experimental models of PM have highlighted promising targets for adjunctive therapy, including cyto- and chemokines, leukocytes, coagulation factors, oxidants, caspases, complement factors, and MMPs [36–39]. To our knowledge, there are no reports so far that have tested adjunctive drugs in experimental MM, except for our published work reporting the beneficial effects of the MMP inhibitor batimastat in a murine model of MM [32]. In humans, the only adjuvant treatment recommended in the current BM guidelines is dexamethasone which is, however, not advised in patients suffering of MM [40]. Therefore, there is an urgent need for adjunctive therapies in MM.

In the present study, we have implemented the aforementioned mouse model [32] to more closely mimic the clinical condition of patients with acute MM. Compared to our former work where adjunctive therapy was started 1 h before infection and a control group treated with CRO was not included [32], here antibiotic and adjuvant treatment were initiated 18 hpi when mice were overtly symptomatic. Results showed that CRO and CRO + DOX were equally effective at increasing mouse survival and reducing brain damage compared to untreated controls, indicating that DOX was not an added value for the clinical outcome of infected mice. Some studies have shown that the impact of adjunctive drugs on brain damage is more pronounced when given as a pretreatment [41, 42] or within the first few h after infection [33, 43] rather than when meningitis is fully developed [9]. Here, we chose to start treatment at 18 hpi on symptomatic animals in the attempt to reproduce the clinical management of patients presenting with acute MM, and this may explain why we failed to show adjunctive activity of DOX in the model.

Several synthetic MMP inhibitors (i.e., batimastat, GM6001, BB1001, TNF-484, Ro 32–7315, Trocade, and DOX) have been tested in experimental PM, and different outcomes were observed in infected animals [12]. Cortical necrosis was consistently attenuated with all inhibitors [18, 22, 33, 41–46], while reduced hippocampal apoptosis [33, 41, 44, 45], increased survival [22, 33, 41, 45, 46], decreased hearing loss [22, 46], and improved learning and memory functions [41, 44, 45, 47] were achieved with specific molecules.

The efficacy of adjuvant DOX was assessed in two separate studies where the molecule was tested alone [22] or in combination with daptomycin [46] in an infant rat model of PM. In both reports, adjunctive DOX proved to be very effective at improving animal survival, reducing injury to brain and cochlea, and diminishing hearing loss compared to rats administered with CRO [22, 46]. In our case, animals in the CRO + DOX group presented improved clinical scores (*p* = 0.0062), increased survival (*p* = 0.017), reduced cerebral hemorrhages (*p* = 0.029), and lower levels of MMP-9 in the brain (*p* = 0.046) in comparison with untreated controls. These results are consistent with our previous work [32] which showed that batimastat significantly diminished brain damage compared to untreated mice. That study, while proving the efficacy of batimastat as an MMP inhibitor in experimental MM, did not assess its value as an adjunctive drug since a treatment group with CRO monotherapy was not included [32]. According to our findings on cerebral bleeding and MMP-9 (Figs. 2 and 3), combined therapy seemed to be more neuroprotective than CRO. However, the slightly larger animal size of CRO + DOX (*n* = 22) compared to CRO (*n* = 18) may have biased the outcome of statistical analysis. Regarding the MMP-9 data, as gelatin zymography measured MMP-9 levels rather than its enzymatic activity, the present results do not allow to infer whether DOX had an impact on MMP-9 activity in vivo. In contrast to the literature on adjuvant DOX in experimental PM [22, 46], in our case no significant differences in clinical parameters, inflammatory mediators and brain damage emerged between CRO and CRO + DOX, suggesting that adjunctive DOX does not offer significant benefits over CRO monotherapy in the MM model.

## Conclusions

Opposite to the proven efficacy of adjunctive DOX in experimental PM, the present data indicate that combined therapy with CRO and DOX does not improve the outcome of MM in mice compared to CRO monotherapy. However, this negative finding is still of importance as it underlines the differences between MM and PM, and reinforces the need of testing novel adjunctive drugs in preclinical studies of MM.

## Data Availability

The datasets used and/or analysed during the current study are available from the corresponding author on reasonable request.

## Abbreviations

BM: Bacterial meningitis
MM: Meningococcal meningitis
PM: Pneumococcal meningitis
MMP: Matrix metalloproteinase
BBB: Blood-brain barrier
CSF: Cerebrospinal fluid
i.c.: Intracisternally
i.p.: Intraperitoneally
s.c.: Subcutaneously
LD_20_: Lethal dose killing 20% of animals
hpi: Hours post infection
PBS: Phosphate buffered saline
CRO: Ceftriaxone
DOX: Doxycycline
CFU: Colony forming units
TNF-α: Tumor necrosis factor α
TACE: TNF-α converting enzyme
IL: Interleukin
MCP-1: Monocyte chemoattractant protein 1
MIP-1β: Macrophage inflammatory protein 1 β
IP10 (CXCL10): Interferon γ-induced protein 10
KC (CXCL1): Keratinocyte chemoattractant
TIMP-1: TIMP metalloproteinase inhibitor
